# The prevalence of myasthenia gravis is increased in inflammatory bowel disease

**DOI:** 10.1055/s-0045-1807717

**Published:** 2025-05-13

**Authors:** Antônio Miguel Furtado Leitão, Florian P. Thomas, Marcellus Henrique Loiola Ponte de Souza, Lúcia Libanez Bessa Campelo Braga, Francisco de Assis Aquino Gondim

**Affiliations:** 1 Universidade Federal do Ceará, Faculdade de Medicina, Departamento de Morfologia, Fortaleza CE, Brazil.; 2 Hackensack University Medical Center, Hackensack Meridian School of Medicine, Department of Neurology, Hackensack NJ, United States.; 3 Universidade Federal do Ceará, Faculdade de Medicina, Departamento de Medicina Clínica, Serviço de Gastroenterologia, Fortaleza CE, Brazil.; 4 Universidade Federal do Ceará, Faculdade de Medicina, Departamento de Medicina Clínica, Serviço de Neurologia, Fortaleza CE, Brazil.

**Keywords:** Crohn Disease, Inflammatory Bowel Diseases, Myasthenia Gravis, Colitis, Ulcerative

## Abstract

**Background:**

Comorbid autoimmune disorders affect approximately 0.2% of the population. A second autoimmune disease occurs in up to 15% of myasthenia gravis (MG) patients.

**Objective:**

To evaluate the association between MG and inflammatory bowel disease (IBD).

**Methods:**

We conducted a cross-sectional study involving a Brazilian cohort of IBD patients and a literature review.

**Results:**

In 2022, we found 1 MG patient with ulcerative colitis and 3 with Crohn's disease out of 606 IBD patients (0.66% prevalence). The patient with UC and MG died in April 2024. The mean IBD onset age was 33.5 ± 2.7; patients were 45.8 ± 7.3-years-old at evaluation. Further, 2 patients were acetylcholine receptor antibody positive, 1 was anti-muscle specific kinase positive, and 1 seronegative. Also, 3 had abnormal repetitive nerve stimulation, all had normal nerve conduction studies, abnormal skin wrinkling test, and mild small fiber neuropathy. None had thymoma and/or underwent thymectomy. According to the MG Foundation's classification, one was class V, one IVb, and two IIa. The MG diagnosis was masked by immunotherapy in all. The prevalence ratio of MG in IBD patients versus the proportion of MG among all patients in our center was 8.56 ( *p* < 0.0001, CI = 3.1–23.5). Considering the lowest and highest prevalence of this condition reported in the literature, the ratio is 44.0 ( *p* < 0.0001, CI: 16.3–118.4) and 26.4 ( *p* < 0.0001, CI: 9.8–70.6), respectively.

**Conclusion:**

The prevalence of MG is higher in IBD, may include muscle specific kinase positive disease (first report in the literature) and frequently overlaps with other autoimmune conditions and small fiber neuropathy.

## INTRODUCTION

Co-occurrence of autoimmune conditions is not rare. A Sardinian survey reported a prevalence of 5.1% of 1 autoimmune disorder, and co-occurrence of 2 of 0.2%. 1 A second autoimmune disease occurs in 15% of myasthenia gravis (MG) patients, especially with early onset and thymic hyperplasia. 2 Early-onset MG is associated with HLA-DR3 and B8, 3 while the late-onset disease with HLA-DR2, B7, and DRB1. 3 This association is higher than in patients with multiple sclerosis (MS). 3

A higher association of MG with autoimmune disorders, especially thyroiditis and rheumatoid arthritis, was confirmed in a Swedish cohort but no link to inflammatory bowel disease (IBD) was established. 3 In one report, thymectomy led to the development of additional autoimmune disorders in MG. 4

Multiple neurological disorders have been detected in IBD patients. 5 6 Since few studies have addressed this subject, we started a cohort study in 2004 to evaluate this issue. 6 A variable prevalence of neurological disorders in IBD has been reported due to different inclusion criteria and ethnic backgrounds. 5 6 Autoimmune diseases reported in IBD include peripheral neuropathy, myelopathy, MS, optic neuritis, and MG. 5 6 Here, we present the epidemiological, clinical, and electrodiagnostic findings of four IBD patients with MG from a 18-year cohort study, as well as a literature review about the subject.

## METHODS

Patients with IBD and neurological diseases seen between 2004 and 2022 at the IBD Clinic of the Hospital Universitário Walter Cantídio, Universidade Federal do Ceará, were invited to participate in a cohort study named NEURODII. After obtaining informed consent, they were evaluated for neurological disorders using a published protocol. 6 In a prior cross-sectional study, 2 IBD patients with MG were found. 7 Here, we describe the clinical findings of all patients diagnosed with MG in this cohort in 2022 (second cross-sectional study), after conducting a statistical analysis to evaluate the risk of MG in IBD versus the prevalence of MG among other patients from our center. The total number of MG patients from our center in 2022 was established by searching the pharmacy databank that lists all the pyridostigmine and immunosuppression medicines prescribed. The total number of IBD patients was provided by the gastroenterology service. Finally, the total number of patients seen over 1-year in our medical center (4.1.21 to 3.31.22) was reported by our record department. These numbers represented the last continuous 1-year period available when we wrote this manuscript.

**Figure 1:**
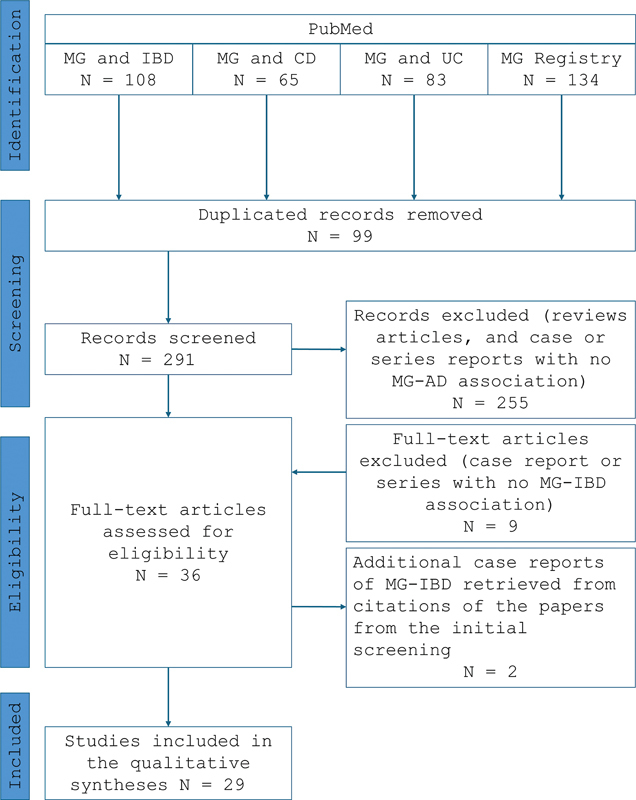
Abbreviations: AD, autoimmune diseases; CD, Crohn's disease; IBD, inflammatory bowel disease; MG, myasthenia gravis; UC, ulcerative colitis. Flowchart of the literature review of patients with MG and IBD.

The MG patients with IBD underwent antibody testing, electromyography/nerve conduction study (EMG/NCS), repetitive nerve stimulation (RNS), and skin wrinkling test for evaluation of small fiber neuropathy. 8 A PubMed search of the literature of all cases of patients with IBD and MG was conducted on January 12, 2023, with the terms “inflammatory bowel disease”, “Crohn's disease” (CD) and “ulcerative colitis” (UC), and “myasthenia gravis” or “myasthenia gravis registry”. All papers were included, regardless of language, provided minimal epidemiological data (e.g. gender, age, disease onset and course) could be extracted, as shown in Figure 1 .

To evaluate the overall risk of MG in IBD, we also included a separate literature review comprising all MG registries that listed patients with other autoimmune diseases, including IBD. Due to the high number of IBD registries in the literature, it was not feasible to evaluate the MG prevalence in all published IBD registries. To our knowledge, no other such report has been published.

### Statistical analysis

A prevalence ratio (PR) based on descriptive statistics (mean ± standard error) and risk assessment of MG versus the total number of patients seen in our center and worldwide was conducted. Statistical analyses were performed using the IBM SPSS Statistics for Windows software, version 20.0. A type-I error probability ( *p* -value) of < 0.05 was assumed.

### Ethical statement

The current study was approved by the Institutional Review Board of Universidade Federal do Ceará (CAAE: 62248416.7.0000.5045) and was conceived according to the principles of the Declaration of Helsinki. All patients invited to participate signed the free and informed consent form.

## RESULTS

In 2022, there were 4 patients with MG in our cohort of 606 IBD patients, with a prevalence of 0.66%, all of whom had been diagnosed by our team over 18-years (three with CD and one with UC). No additional patients were diagnosed over this period (no follow-up loss). At evaluation, patients were 45.75 ± 7.29-years-old. Mean age at IBD diagnosis was 33.5 ± 2.69, and mean age at MG onset was 39.5 ± 2.69. All patients had MG diagnosed after several years of IBD involvement. There were three women and one man. Preliminary results of two cases were reported elsewhere. 7 The PR of MG in IBD patients (54/70,089) versus the proportion of MG among all patients in our center (4/606) was of 8.56 ( *p* < 0.0001; CI = 3.1–23.5).

### Patient 1 (initial findings previously reported) 7

A 37-year-old man was diagnosed with UC but 3 years later changed to CD after developing gastrocolonic fistula following a colectomy for colonic dysplasia. Assuming that he was cured, he missed follow-up evaluations and stopped prednisone and azathioprine.

He developed quadriparesis with bilateral ptosis, dysphagia and dysarthria. Upon admission, he required mechanical ventilation. Following this, MG was diagnosed; the EMG/NCS results were normal. Right ulnar RNS revealed basal compound muscle action, with potential decrements of up to 22% that improved after exercise (correction) and increased 2 minutes after forced muscle contraction, up to 31%. Acetylcholine receptor antibodies (AchRAB) were elevated to 10.7. The P-ANCA titers were positive (1:640), and TSH decreased (0.2).

Since discharge after diagnosis, MG remained well controlled with the prednisone (tapered to 5 mg QD, over the last 7 years), azathioprine (100 mg QD) and pyridostigmine (60 mg every 4h), except for occasional fatigability. Since diagnosis, he never had a myasthenic crisis or required additional treatments. He had no thymoma and declined thymectomy. Small fiber neuropathy was diagnosed, based on distal paresthesia and abnormal skin wrinkling test.

### Patient 2 (initial findings previously reported) 7

A 35-year-old woman was diagnosed with UC. Subsequent development of jaundice, anemia & hepatosplenomegaly led to a diagnosis of primary sclerosing cholangitis. After 5 years, she developed speech impairment, bilateral ptosis, disconjugate gaze, fatigability with sustained vertical movements and mild proximal arm weakness. The EMG/NCS results were normal. Right ulnar RNS revealed significant decrement with correction after exercise and worsening three minutes post exercise. Mild sensory findings and abnormal skin wrinkling (mean score: 0.5) were consistent with small fiber neuropathy. Furthermore, AchRAB were above 20, antinuclear antibody (ANA) was positive, and immunoglobulin G-4 (IgG4) level was normal.

Initial treatment with pyridostigmine and prednisone was successful. Thymectomy could not be performed due to thrombocytopenia (idiopathic thrombocytopenic purpura vs. myelodysplastic syndrome). She was treated with prednisone and intravenous immunoglobulin (IVIG), azathioprine being contraindicated due to cirrhosis.

In August 2021 she was started on rituximab with good MG control. In February 2023, she was diagnosed with heart failure, pulmonary hypertension due to severe mitral and moderate tricuspid insufficiency (possible sequelae of rheumatic fever), and moderate pericardial effusion. After 1 month, she was admitted with multiple medical problems and experienced MG worsening. She was treated with IVIG with MG improvement, but died on April 2023 due to multiple medical complications: worsening cirrhosis, congestive heart failure, pneumonia, and septic shock.

### Patient 3

A 37-year-old woman was diagnosed with CD after a 10-year history of diarrhea, weight loss, and abdominal pain. A colonoscopy done in 2026 revealed aphthous ileal, cecal, and rectal ulcers. She was treated with sulfasalazine and mesalazine but then developed a perianal fistula. Azathioprine treatment began in May 2019 but stopped due to abdominal pain and elevated liver enzymes.

Upon neurological evaluation of headache, she received a diagnosis of trigeminal neuralgia. She also complained of dizziness, distal paresthesias, and recurrent left facial palsy. With a previous diagnosis of fibromyalgia, she met the diagnostic criteria of restless leg syndrome and small fiber neuropathy with an abnormal skin wrinkling (mean score: 1.75). On exam she had bilateral (predominant left) facial weakness, hyperreflexia with bilateral Hoffmann sign, and distal pin prick loss.

In July 2021, based on clinical findings and abnormal brain magnetic resonance imaging (MRI), she was diagnosed with MS and started on glatiramer acetate. Brain MRI did not disclose brainstem involvement. She was anemic (hemoglobin = 7.2) and had borderline low vitamin B12, at 227.

In May 2022, the facial weakness worsened with disconjugate eye movements and fatigability on sustained upgaze, mild asymmetric ptosis (worse in the late afternoon). She was diagnosed with MG. The EMG/NCS and RNS results were normal; AchRAB were negative; and antimuscle-specific tyrosine kinase (anti-MUSK) could not be done.

She improved with pyridostigmine and prednisone (40 mg/day). Prednisone was later tapered to 10 mg alternating with 20 mg/day, but since it could not be reduced any further due to worsening eye findings and fatigability, rituximab is currently under consideration (not started due to limited institutional availability). Chest computed tomography (CT) showed no thymoma and thymectomy was declined.

### Patient 4

A 37-year-old woman was diagnosed with CD after an 8-year history of bloody diarrhea and 17kg weight loss and abnormal colonoscopy. She was initially treated with mesalazine and prednisone and then azathioprine. After developing an anovaginal fistula, inflixamab was started. She developed episodic right eye pain and migraine 20 days prior to inflixamab infusions

In April 2021, she developed left ophthalmoplegia and vision loss. She was admitted for inpatient treatment with intravenous (IV) methylprednisolone after being diagnosed with orbital apex syndrome due to posterior orbital pseudotumor. Vision loss resolved with steroids but ophthalmoplegia persisted, and she was evaluated for MG. The EMG/NCS results were normal; RNS disclosed a greater than 10% facial nerve decrement; AchRAB were negative; and anti-MUSK antibodies were elevated (0.09 NMOL/L); aquaporin and thyreoglobulin antibodies were normal; IgG4 levels were normal; vitamin B12 was low (173) and treated; chest CT showed no thymoma.

She responded to pyridostigmine and prednisone 40 mg/day. Prednisone was tapered off and, since last year (2024), MG has been controlled (no neurological signs) with azathioprine 100 mg/day. She had mild distal paresthesias consistent with small fiber neuropathy, confirmed by abnormal skin wrinkling (mean score: 0.5).

### Literature review

As shown in Table 1 and Figure 1 , our literature review disclosed 23 papers and 30 patients with IBD and MG. 3 4 5 7 9 10 11 12 13 14 15 16 17 18 19 20 21 22 23 24 25 26 27 28 29 30 31 32 33 34 Some articles did not provide important demographic details. Seven additional papers describing MG registries where additional autoimmune diseases could be identified were also evaluated.

**Table 1:** Literature review of all cases reports, case series, and registries of myasthenia gravis and inflammatory bowel disease

Country	Gender	Age ^‡^	Interv ^§^	MGF	Abs	Neur. Dx	Thymec.	Thymec. impact	Thym.	MGE/C	Immuno MG Tx	Other AD/Ca	IBD status
**Crohn's disease**													
Israel 5	M	11	9	≥II	AchR	n/a	n/a	n/a	n/a	n/a	n/a	n/a	n/a
Brazil 7 Italy 9	M F	37 68	3 –(?)	V IIA?	AchR n/a	RNS+ n/a	No n/a	n/a n/a	No n/a	Yes No	Pred; Azat Pred; Azat + Pred	SFN; Hyperthyr. RA, RU, gallstone	Mild Moderate
UK 10	F	19	3	I	AchR	Edro +	Yes	MG & CD (↑)	No	No	Pred	Epis, EN	Moderate at onset and then worsened
Italy 11	F	26	–4	n/a	n/a	n/a	Yes	No	n/a	No	Pred	Thyroiditis, HT, EN	Mild
USA 12	M	48	15	IIb	AchR (15.00)	RNS –; Edro +	No	No	no	Yes	Pred + Azat; Plas	n/a	Moderate
USA 13	F	26	n/a	n/a	n/a	n/a	n/a	n/a	n/a	n/a	Psol +IVIg + Ecul; Psol + Azat	JRA	Moderate
India 14	M	50	12	IV	AchR (13.46)	Edro +	Yes	MG (↑↑)	yes	n/a	Pred	n/a	Mild
India 15	M	57	5	IIa	AchR (13.46)	n/a	Yes	n/a	yes	No	Azat; Steroids	n/a	In remission
Romania 16	n/a	n/a	n/a	I	AchR	n/a	n/a	n/a	n/a	n/a	n/a	n/a	n/a
Romania 16	n/a	n/a	n/a	I	AchR	n/a	n/a	n/a	n/a	n/a	n/a	n/a	n/a
**Crohn's disease**													
Japan 17	F	60	–2	n/a	AchR (34.00)	n/a	Yes	n/a	n/a	No	Ambc	Thyroid Ca.	Severe
**Ulcerative colitis**													
USA 4	F	15	–4	IVa	n/a	RNS +; Edro +	Yes	MG (↑↑↑); SLE & UC onset	n/a	Yes	No	SLE, cirrhosis	Severe
Romania 16	n/a	n/a	n/a	I	AchR	n/a	n/a	n/a	n/a	n/a	n/a	n/a	n/a
Romania 16	n/a	n/a	n/a	I	AchR	n/a	n/a	n/a	n/a	n/a	n/a	n/a	n/a
Romania 16	n/a	n/a	n/a	I	negative	n/a	n/a	n/a	n/a	n/a	n/a	n/a	n/a
Brazil 7 USA 18	F M	35 14	5 7	IVa ≥IIIb	AchR AchR	RNS + n/a	n/a n/a	n/a n/a	n/a n/a	Yes Yes	Pred Plas; Azat + Pred	PSCç SFN FDGS, PSC	Mild In remission
France 19	F	17	10	IIIb	AchR (20.00)	RNS +	n/a	n/a	n/a	No	No	Sacroiliitis, DVT	Mild at onset and then worsened
Sweden 20	F	26	5	IIIb	n/a	n/a	Yes	MG (↑↑↑); UC (↑↑)	Yes	No	No	n/a; Mot: RA; Sister: SLE	Mild
Sweden 20	F	23	–1	IIIb	AchR (1.00)	n/a	Yes	MG (↑↑)	no	No	No	n/a	Mild
Turkey 21	F	21	10	IIIb	AchR (2.00)	RNS –	n/a	n/a	n/a	No	Steroids	n/a	Severe
UK 22	M	77	2	IVb	AchR (5.21)	EMG/SF+	n/a	n/a	n/a	No	Psol + IVIg; Myco	PBC, vitiligo, SM PN	Mild
UK 23	M	35	13	V	Negative	n/a	n/a	n/a	n/a	Yes	Tetr; Pred	LP	Mild
UK 24	M	n/a	n/a	n/a	n/a	n/a	Yes	n/a	n/a	n/a	n/a	HT, colon Ca	Severe
UK 24	M	n/a	n/a	n/a	n/a	n/a	n/a	n/a	n/a	n/a	Pred + Azat	n/a	Severe
UK 25	M	34	12	V	negative	Edro +	n/a	n/a	n/a	Yes	Steroids	LP, AA, vitiligo	Mild
UK 26	F	40	0.5	IVa?	AchR (12.07) Musk –	n/a	Yes	MG & UC (↑↑↑)	Yes	Yes	Plas + Ster + Azat	Retinopathy	Mild
Japan 27	M	19	7	V?	AchR (6.30)	n/a	Yes	MG & UC (↑↑)	No	No	Pred + Cicl; IVIg	AT, GCM	Mild but severe EIM
France 28	M	14	0.2	IIIb	AchR (13.80)	RNS +	n/a	n/a	n/a	No	Azat	KS, AH + SC; Father: AS; Mother: GD + RA	Moderate
**Country**	**Study design – MG registry**		**Sample size**		**MG + IBD** **Prevalence (%)**		**RP and/or FU**		**Demographic factors**				
Italy 29	Poly-autoimmunity in MG patients		984		5 (0.50%)		FU: 2–40 years		All MG + IBD patients are AchR+ and GMG: 2 early onset, 2 late onset, 1 with thymoma				
UK 30	Clinical burden in a MG cohort		1.149		19 (1.65%)		RP: 1997–2016 (20 years)		All MG + IBD patients are non-refractory to treatment. 7 have CD and 12 UC. (According to the paper, 5 -15% of the MG patients are refractory to conventional treatment)				
Netherlands 31	Patient registry of MG and L-E myasthenic syndrome		565		2 (0.35%)		RP: 12/2015 - 9/2019		Thymectomy patients: 53%. In the online search: 47% of the patients don't know the type of antibody that they have (In the Dutch registry, only 6-7% don't have the antibody results)				
Sweden 32	Concomitant autoimmunity in MG		547		1 (1.82%)		n/a		Thymectomy patients: 59.5%.				
Australia 33	Clinical features and impact of MG		165		3 (1.81%)		n/a		Thymectomy patients: 30.2% (39/165). MG remission = 55.5% (20/39) of the thymectomy patients				
USA 34	OMG in a senior population		39		1 (2.56%)		RP: 1998–2007		Among the OMG that did not evolve to GMG, 52% received immunotherapy. In 2 patients, MG started before the AD (8AD/39MG)				
Sweden 3	MG autoimmune spectrum: a Swedish population-based study		2,045		39 (1.90%)		RP: Born between 1932–2002 FU: 7/1/2005–12/31/2010		MG + CD = 18; OR = 2.3 (for MG and CD). MG + UC = 21; OR = 2.1 (for MG and UC). Thymoma: 62/2045 = 7.9%				

Abbreviations: AA, alopecia areata; ABs, antibodies; AchR, anti-acetylcholine receptor; AD/Ca, autoimmune disease/cancer; AH, autoimmune hepatitis; Ambc, ambenonium chloride; AS, ankylosing spondylitis; AT, atopic dermatitis; Azat, azathioprine; CD, Crohn's disease; Cicl, cyclosporine; DVT, deep vein thrombosis; Ecul, eculizumab; Edro +, edrophonium test positive; EIM, extraintestinal manifestation; EMG/SF +, electromyography single fiber increased jitter; EN, erythema nodosum; Epis, episcleritis; FSGS, focal segmental glomerular sclerosis; FU, follow-up; GCM, giant cell myocarditis; GD, Grave's disease; GMG, generalized myasthenia gravis; HT, hypothyroidism; Hyperthyr, hyperthyroidism; IBD, inflammatory bowel disease; Immuno MG Tx, immune treatment of MG; IVIG, immunoglobulin IV; JRA, juvenile rheumatoid arthritis; KS, Kawasaki syndrome; L-E, Lambert Eaton; LP, lichen planus; MG, myasthenia gravis; MGE/C, myasthenia gravis exacerbation/crisis; MGF, MG foundation's classification; Myco, mycophenolate; Musk, anti-Musk; negative, seronegative; Neur. Dx, neurophysiological diagnosis; OMG, ocular myasthenia gravis; OR, odds ratio; PBC, primary biliary cirrhosis; Plas, plasmaferese; Pred, prednisone; PSC, primary sclerosing cholangitis; Psol, prednisolone; RA, rheumatoid arthritis; RNS-, repetitive nerve simulation without decrement; RNS + , repetitive nerve simulation with decrement; RP, registration period; RU, recurrent uveitis; SC, sclerosing cholangitis; SFN, small fiber neuropathy; SLE, systemic lupus erythematosus; SM PN, sensorimotor axonal peripheral neuropathy; Ster, steroids; Tetr, tetracosactrin; Thym, thymoma; Thymec., thymectomy; UC, ulcerative colitis; UK, Uniyed Kingdom; USA, United States of America.

Notes: ^‡^ Age of diagnosis of IBD; ^§^ time interval between IBD and MG diagnosis (in years); (↑) improvement; (↑↑) major improvement; (↑↑↑) remission.

There were 12 patients (5 women, 5 men, and 2 of unknown gender) with MG and CD, mean age of CD diagnosis was 40.2 ± 18.2 years. In one included case, the CD diagnosis was not clearly defined at that time. There were 18 patients (8 men, 7 women, 3 unknown gender) with UC and MG, mean age of UC diagnosis 28.5 ± 16.4 years. Most often, the MG diagnosis preceded the IBD diagnosis, 3 to 15 years for CD and 0.2 to 13 years for UC. In two UC and three CD patients, MG was diagnosed 1 to 4 years prior to IBD diagnosis (one had undefined duration). There were 16 patients with additional autoimmune diseases, 5 for CD and 11 for UC. Finally, one patient had thyroid cancer, and another colon cancer.

According to the MG Foundation's classification, 30% were class IV and V. Pure ocular disease (I) and moderate forms (IIIb) occurred in the same proportion (6/25, 24% each), milder forms of generalized disease (II) affected 16% of the patients. Among 23 patients who underwent antibody testing, 20 (86.9%) were AchRAB positive, and 3 negative (13.04%). Only one had MUSK antibody testing which was negative. None were tested for lipoprotein receptor related protein-4 (LRP4) or agrin antibodies.

Thymectomy led to improvement of MG in 54.5% of patients (6/11), but in one patient in MG remission, systemic lupus erythematosus (SLE) and UC started after thymectomy. Furthermore, four cases lacked information about thymectomy outcome. Among the seven MG registries detailing associated autoimmune conditions, the prevalence of co-occurring IBD varied from 0.35% in 565 MG patients, reaching up to 2.56% in the 39 patients older than 70 years.

## DISCUSSION

Major gaps remain about the disease mechanisms involved in the extraintestinal manifestations of IBD. Additional autoimmune disorders affect up to 9.4% of UC patients, 16 and up to 15% of MG patients. 2 In our patients with IBD and MG, all had at least a third additional autoimmune disorder: orbital pseudotumor, primary sclerosing cholangitis, idiopathic thrombocytopenic purpura, hyperthyroidism, MS, and small fiber neuropathy.

The prevalence of MG was 0.66% in the present cross-sectional study. This prevalence is eight times higher than in a cohort of rheumatoid arthritis patients 35 and maybe within the same range of patients with a SLE of 0.15 to 1.3%. 36 A Swedish MG registry study documented a 2.3 times increased risk of CD in MG patients (CI = 1.3–4.0) and 2.1 times increased risk for UC (CI = 1.3–3.5). 3 Our study is the first to provide consistent statistical evidence for increased risk of MG in IBD patients. The prevalence ratio (PR) of MG in IBD versus its proportion among all patients seen in our center was 8.56 ( *p* < 0.0001, CI = 3.1–23.5). Considering the lowest and highest prevalence of MG in the literature the PR is even higher: 44.00 ( *p* < 0.0001, CI: 16.3–118.4) and 26.40 ( *p* < 0.0001, CI: 9.8–70.6), respectively.

What are the possible explanations for this association? Although we were not able to test it, both MG and IBD are associated with certain HLA genotypes. 3 They both feature abnormalities in innate and adaptative immunity. 3 5 6 A pro-inflammatory environment and imbalance in T-regulatory and T-helper-17 lymphocytes are also present. 16 37 Increased production of chemokine C-X-C ligand-13 may be another explanation. 37 Common abnormalities in the gut–brain axis may play a role, since the thymus receives major vagal innervation. 38

Furthermore, MG and IBD are complex diseases, with different subtypes and pathomechanisms. First, MG can be caused by different mechanisms and types of antibodies: AchRAB, MUSK, LRP4, and agrin antibodies. 29 Anti-MUSK disease is mediated by IgG4 antibodies, and the role of LRP4 and agrin antibodies are less studied. Then there are seronegative subtypes of MG, for which different mechanisms are likely involved. On the other hand, there are two subtypes of autoimmune pancreatitis that are considered extraintestinal manifestations of IBD. 39 Type II, the most prevalent, may share pathomechanisms with anti-AchRAB MG, with overexpression of cellular adhesion molecule Mad-1. 39 Type I is part of the spectrum of IgG4 diseases, similar to anti-MUSK MG. 39 In fact, up to 4% of the IBD patients can have increased IgG4 levels. 39

To our knowledge, our Patient 4 is the first reported anti-MUSK MG in IBD. Her disease course is unusual, with orbital pseudotumor, a rare extra-intestinal manifestation of IBD. The presence of SOX1 antibodies in a patient with CD and Lambert-Eaton myasthenic syndrome points towards a wider association of different types of neuromuscular involvement. 40

Our literature review disclosed 23 papers and 30 patients with IBD and MG, as well as 7 MG registries detailing IBD patients ( Table 1 ). 3 5 7 9 10 11 12 13 14 15 16 17 18 19 20 21 22 23 24 25 26 27 28 29 30 31 32 33 34 The onset age of MG resembled the early peak in non-IBD patients. Whereas females predominated in CD patients, men outnumbered women with UC and MG. Close to 90% were AchRAB positive. However, since most papers predated older anti-MUSK description, only one had a negative test. None was tested for LRP4 or agrin antibodies. Thymectomy led to improvement of MG in the majority. Similar to our patients, many additional autoimmune diseases were found.

In our study, patient 1 had hyperthyroidism, and 2 had primary sclerosing cholangitis in addition to MG and UC, a triple association that has been reported only once before. 18 Patient 3 had MG, CD, and MS, and patient 4 had anti-MUSK MG, CD, and orbital pseudotumor, which are triple associations never reported previously. In addition, our four patients had subtle small fiber neuropathy and low-borderline B12 levels. The small fiber neuropathy could have partially resulted from B12 deficiency, which is frequently autoimmune), but immune-mediated small fiber neuropathy cannot be excluded. Furthermore, patient 1 illustrates that immunosuppression for IBD may mask MG and lead to misdiagnosis. However, prednisone, azathioprine, and infliximab may have also delayed the diagnosis in patients 2, 3, and 4, due to partial or even complete control of MG. This pattern has been reported previously. 15 26 27

None of our patients underwent thymectomy. Reasons included patient refusal and persistent thrombocytopenia. In our literature review, thymectomy usually produced good results, occasionally improving both IBD and MG. However, we noted at least one patient in whom SLE and UC started after thymectomy. 4 Two patients experienced total regression of MG after colectomy, 16 19 while three developed MG after colectomy. 21 24 Pyridostigmine did not exacerbate gastrointestinal symptoms in our patients, similar to most other reports.

In conclusion, MG prevalence was of 0.66% in this cross-sectional study involving our cohort of IBD patients. This prevalence is at least 8 times higher than in the other patients seen in our center, and 26 to 44 times higher than in the general population worldwide. The spectrum of MG in IBD may include anti-MUSK positive disease. In general, the clinical course of this disease was not significantly modified by IBD relapses. Therefore, MG needs to be considered in IBD patients with new onset ocular, bulbar, or limb symptoms, in particular after changes in immunosuppression. Further studies including larger cohorts or national registries are necessary to confirm this association.

## References

[1] Sardu C Cocco E Mereu A Massa R Cuccu A Marrosu M G Contu P Population based study of 12 autoimmune diseases in Sardinia, Italy: prevalence and comorbidity PLoS One 2012 7 03 e32487 10.1371/journal.pone.0032487 22396771 PMC3292563

[2] Gilhus N E Myasthenia Gravis N Engl J Med 2016 375 26 2570 2581 10.1056/NEJMra1602678 28029925

[3] Fang F Sveinsson O Thormar G Granqvist M Askling J Lundberg I E The autoimmune spectrum of myasthenia gravis: a Swedish population-based study J Intern Med 2015 277 05 594 604 10.1111/joim.12310 25251578

[4] Galbraith R F Summerskill W H Murray J Systemic lupus erythematosus, cirrhosis and ulcerative colitis after thymectomy for myasthenia gravis N Engl J Med 1964 270 229 231 10.1056/NEJM196401302700504 14072077

[5] Lossos A River Y Eliakim A Steiner I Neurologic aspects of inflammatory bowel disease Neurology 1995 45(3 Pt 1):416 421 10.1212/wnl.45.3.416 7898687

[6] Gondim FdA Oliveira GRd Teles B C Souza M H Braga L L Messias E L A case-control study of the prevalence of neurological diseases in inflammatory bowel disease (IBD) Arq. Neuro-Psiquiatr. 2015 73 02 119 124 10.1590/0004-282 × 20140223 25742581

[7] Gondim FdA Oliveira GRd Araújo D F Souza M H Braga L L Thomas F P Two patients with co-morbid myasthenia gravis in a Brazilian cohort of inflammatory bowel disease Neuromuscul Disord 2014 24 11 999 1002 10.1016/j.nmd.2014.06.434 25065584

[8] Gondim FdA Araújo D F Oliveira I S Vale O C Small fiber dysfunction in patients with Wilson's disease Arq. Neuro-Psiquiatr. 2014 72 08 592 595 10.1590/0004-282 × 20140090 25003396

[9] Angelucci E Cesarini M Vernia P Successful resolution of pneumonia developed in a patient affected by Crohn's disease, rheumatoid arthritis, myasthenia gravis and recurrent uveitis during concomitant treatment with tumour necrosis factor alpha inhibitors and conventional immunosuppressive drugs Rheumatol Int 2010 30 07 977 978 10.1007/s00296-009-1012-6 19551384

[10] Finnie I A Shields R Sutton R Donnelly R Morris A I Crohn's disease and myasthenia gravis: a possible role for thymectomy Gut 1994 35 02 278 279 10.1136/gut.35.2.278 8307484 PMC1374509

[11] Manfredi R Fasulo G Fulgaro C Sabbatani S Associated thyreoiditis, myasthenia gravis, thymectomy, Chron's disease, and erythema nodosum: pathogenetic and clinical correlations, immune system involvement, and systemic infectious complications Rheumatol Int 2008 28 11 1173 1175 10.1007/s00296-008-0579-7 18389238

[12] Martin R W Shah A Myasthenia gravis coexistent with Crohn's disease J Clin Gastroenterol 1991 13 01 112 113 2007731

[13] Murphree J Mulherin D W Morton C Adams D High-dose vitamin C therapy for symptomatic deficiency in a patient with myasthenia gravis and Crohn's disease Nutr Clin Pract 2022 37 05 1242 1245 10.1002/ncp.10800 34784069

[14] Kumar S Sultania M Vatsal S Sharma M C Primary Ectopic Mediastinal Goiter in a Patient With Crohn's Disease Presenting as Myasthenia Gravis Ann Thorac Surg 2015 100 06 2333 2336 10.1016/j.athoracsur.2015.02.116 26652525

[15] Garg R Hasija N Thymectomy in a patient with myasthenia gravis and Crohn's disease – Anaesthetic Challenges J Anesth Clin Care 2015 2 01 1 310.24966/ACC-8879/100007

[16] Cojocaru I M Cojocaru M Tănăsescu R Burcin C Atanasiu A N Mitu A C Some clinico-immunological aspects in patients with ocular myasthenia gravis associated with inflammatory bowel disease Rom J Intern Med 2008 46 02 165 168 19284089

[17] Takechi K Mihara M Saito Y Yamada T Moriwaki H Muto Y A case of inflammatory bowel disease accompanied by myasthenia gravis Dig Endosc 1993 5 01 102 106 10.1111/j.1443-1661.1993.tb00601.x

[18] Foroozan R Sambursky R Ocular myasthenia gravis and inflammatory bowel disease: a case report and literature review Br J Ophthalmol 2003 87 09 1186 1187 10.1136/bjo.87.9.1186 12928296 PMC1771868

[19] Gower-Rousseau C Reumaux D Bellard M Delecourt L Ribet M Colombel J F Remission of myasthenia gravis after proctocolectomy in a patient with ulcerative colitis Am J Gastroenterol 1993 88 07 1136 1138 8317426

[20] Hertervig E Nilsson A [Myasthenia gravis in patients with ulcerative colitis–an overlooked autoimmune association?] Lakartidningen 1992 89 36 2860 2862 1405884

[21] Koc F Yerdelen D Y Rare association of myasthenia gravis and ulcerative colitis Neurosciences (Riyadh) 2009 14 04 382 383 21048657

[22] McCann P Pramanik A Dysphagia and unexpected myasthenia gravis associated with primary biliary cirrhosis, ulcerative colitis and vitiligo J Am Geriatr Soc 2004 52 08 1407 1408 10.1111/j.1532-5415.2004.52379_7.x 15271143

[23] Miller T N Myasthenia gravis, ulcerative colitis and lichen planus Proc R Soc Med 1971 64 08 807 808 4328085 10.1177/003591577106400804 PMC1811999

[24] Setti-Carraro P Ritchie J K Wilkinson K H Nicholls R J Hawley P R The first 10 years' experience of restorative proctocolectomy for ulcerative colitis Gut 1994 35 08 1070 1075 10.1136/gut.35.8.1070 7926908 PMC1375057

[25] Tan R S Ulcerative colitis, myasthenia gravis, atypical lichen planus, alopecia areata, vitiligo Proc R Soc Med 1974 67 03 195 196 4820815 10.1177/003591577406700308 PMC1645369

[26] Sanghi P Bremner F An unusual presentation of thymoma: dysgeusia, ulcerative colitis, keratoconjunctivitis sicca, autoimmune retinopathy and myasthenia gravis BMJ Case Rep 2022 15 01 e246861 10.1136/bcr-2021-246861 PMC8762128 35027385

[27] Yagi N Watanabe T Ikeda Y Fukushima N Successful bridge to recovery in a patient with fulminant giant cell myocarditis that developed from multiple autoimmune disorders including myasthenia gravis: a case report Eur Heart J Case Rep 2022 6 02 ytac046 10.1093/ehjcr/ytac046 35198850 PMC8859629

[28] Guinet-Charpentier C Bilbault C Kennel A Perrier P Peyrin-Biroulet L Morali A Unusual association of myasthenia gravis and ulcerative colitis in a 14-year-old boy Arch Pediatr 2015 22 01 81 83 10.1016/j.arcped.2014.10.014 25440769

[29] Evoli A Caliandro P Iorio R Alboini P E Damato V LaTorre G Poly-autoimmunity in patients with myasthenia gravis: A single-center experience Autoimmunity 2015 48 06 412 417 10.3109/08916934.2015.1031890 25868386

[30] Harris L Graham S MacLachlan S Exuzides A Jacob S A retrospective longitudinal cohort study of the clinical burden in myasthenia gravis BMC Neurol 2022 22 01 172 10.1186/s12883-022-02692-4 35534810 PMC9082838

[31] Ruiter A M Strijbos E Meel RHPd Lipka A F Raadsheer W F Tannemaat M R Verschuuren J JGM Accuracy of patient-reported data for an online patient registry of autoimmune myasthenia gravis and Lambert-Eaton myasthenic syndrome Neuromuscul Disord 2021 31 07 622 632 10.1016/j.nmd.2021.05.006 34210541

[32] Ramanujam R Piehl F Pirskanen R Gregersen P K Hammarström L Concomitant autoimmunity in myasthenia gravis--lack of association with IgA deficiency J Neuroimmunol 2011 236(1-2):118 122 10.1016/j.jneuroim.2011.05.008 21669464 PMC3230785

[33] Blum S Lee D Gillis D McEniery D F Reddel S McCombe P Clinical features and impact of myasthenia gravis disease in Australian patients J Clin Neurosci 2015 22 07 1164 1169 10.1016/j.jocn.2015.01.022 26021730

[34] Allen J A Scala S Jones H R Ocular myasthenia gravis in a senior population: diagnosis, therapy, and prognosis Muscle Nerve 2010 41 03 379 384 10.1002/mus.21555 19918767

[35] Bixio R Bertelle D Pistillo F Pedrollo E Carletto A Rossini M Viapiana O Rheumatoid arthritis and myasthenia gravis: a case-based review of the therapeutic options Clin Rheumatol 2022 41 04 1247 1254 10.1007/s10067-022-06062-w 35031874 PMC8913445

[36] Raut S Reddy I Sahi F M Masood A Malik B H Association Between Systemic Lupus Erythematosus and Myasthenia Gravis: Coincidence or Sequelae? Cureus 2020 12 06 e8422Published Jun 3 . 10.7759/cureus.8422 32642338 PMC7336596

[37] Evoli A Meacci E An update on thymectomy in myasthenia gravis Expert Rev Neurother 2019 19 09 823 833 10.1080/14737175.2019.1600404 30917699

[38] Günther C Rothhammer V Karow M Neurath M Winner B The Gut-Brain Axis in Inflammatory Bowel Disease-Current and Future Perspectives Int J Mol Sci 2021 22 16 8870 10.3390/ijms22168870 34445575 PMC8396333

[39] Massironi S Fanetti I Viganò C Pirola L Fichera M Cristoferi L Systematic review-pancreatic involvement in inflammatory bowel disease Aliment Pharmacol Ther 2022 55 12 1478 1491 10.1111/apt.16949 35505465 PMC9322673

[40] Polilli E Frattari A Esposito J E Angelini G Di Risio A Mazzotta E SOX-1 antibodies in a patient with Crohn's disease: a case report BMC Neurol 2022 22 01 404 10.1186/s12883-022-02923-8 36324062 PMC9628059

